# Increased Tumor Intrinsic Growth Potential and Decreased Immune Function Orchestrate the Progression of Lung Adenocarcinoma

**DOI:** 10.3389/fimmu.2022.921761

**Published:** 2022-07-01

**Authors:** Yue Zhao, Jun Shang, Jian Gao, Han Han, Zhendong Gao, Yueren Yan, Qiang Zheng, Ting Ye, Fangqiu Fu, Chaoqiang Deng, Zelin Ma, Yang Zhang, Difan Zheng, Shanbo Zheng, Yuan Li, Zhiwei Cao, Leming Shi, Haiquan Chen

**Affiliations:** ^1^ Department of Thoracic Surgery and State Key Laboratory of Genetic Engineering, Fudan University Shanghai Cancer Center, Shanghai, China; ^2^ Institute of Thoracic Oncology, Fudan University, Shanghai, China; ^3^ Department of Oncology, Shanghai Medical College, Fudan University, Shanghai, China; ^4^ State Key Laboratory of Genetic Engineering, Human Phenome Institute, School of Life Sciences and Shanghai Cancer Center, Fudan University, Shanghai, China; ^5^ International Human Phenome Institutes (Shanghai), Shanghai, China; ^6^ Department of Pathology, Fudan University Shanghai Cancer Center, Shanghai, China; ^7^ School of Life Sciences, Fudan University, Shanghai, China

**Keywords:** imbalance, tumor intrinsic growth potential, immune response, progression, lung adenocarcinoma

## Abstract

**Background:**

The overall 5-year survival of lung cancer was reported to be only ~15%, with lung adenocarcinoma (LUAD) as the main pathological subtype. Before developing into invasive stages, LUAD undergoes pre-invasive stages of adenocarcinoma *in situ* (AIS) and minimally invasive adenocarcinoma (MIA), where surgical resection gives an excellent 5-year survival rate. Given the dramatic decline of prognosis from pre-invasive to invasive stages, a deeper understanding of key molecular changes driving the progression of LUAD is highly needed.

**Methods:**

In this study, we performed whole-exome sequencing and RNA sequencing on surgically resected 24 AIS, 74 MIA, 99 LUAD specimens, and their adjacent paired normal tissues. Survival data were obtained by follow-up after surgery. Key molecular events were found by comparing the gene expression profiles of tumors with different stages. Finally, to measure the level of imbalance between tumor intrinsic growth potential and immune microenvironment, a tumor progressive (TP) index was developed to predict tumor progression and patients’ survival outcome and validated by external datasets.

**Results:**

As tumors progressed to more invasive stages, they acquired higher growth potential, mutational frequency of tumor suppressor genes, somatic copy number alterations, and tumor mutation burden, along with suppressed immune function. To better predict tumor progression and patients’ outcome, TP index were built to measure the imbalance between tumor intrinsic growth potential and immune microenvironment. Patients with a higher TP index had significantly worse recurrence-free survival [Hazard ratio (HR), 10.47; 95% CI, 3.21–34.14; p < 0.0001] and overall survival (OS) [Hazard ratio (HR), 4.83e8; 95% CI, 0–Inf; p = 0.0013]. We used The Cancer Genome Atlas (TCGA)-LUAD dataset for validation and found that patients with a higher TP index had significantly worse OS (HR, 1.10; 95% CI, 0.83–1.45; p = 0.048), demonstrating the prognostic value of the TP index for patients with LUAD.

**Conclusions:**

The imbalance of tumor intrinsic growth potential and immune function orchestrate the progression of LUAD, which can be measured by TP index. Our study provided new insights into predicting survival of patients with LUAD and new target discovery for LUAD through assessing the imbalance between tumor intrinsic growth potential and immune function.

## Introduction

Lung cancer is one of the deadliest disease worldwide, with a 5-year survival of only ~19% (1). Lung adenocarcinoma (LUAD) is the most common pathological subtype. Pre-invasive stages of LUAD, namely, adenocarcinoma *in situ* (AIS) and minimally invasive adenocarcinoma (MIA), have a nearly 100% 5-year survival rate after complete surgery (2, 3). The prognosis of patients would downslide dramatically once the disease progressed to invasive stages. Therefore, it is necessary to study the evolution of LUAD for discovering new targets and developing new treatments. Although there have been studies on genomic and immune profiling of patients with AIS, MIA, and LUAD, there lack a systematic study focusing on key molecular events that drive the evolution of LUAD (4–8).

As tumors progress, their radiological manifestations change. With the development of thoracic computed tomography (CT) scanning and the application of low-dose CT screening, an increasing number of small pulmonary nodules, especially subsolid nodules have been detected (9–12). The prognostic impact of solid components for LUADs presented as ground-glass opacities (GGOs) on CT scanning has been under extensive investigation, where tumors manifesting as GGOs were found to have indolent clinical course (13, 14). Our two previous studies further provided evidence on the prognostic value of LUADs, manifesting as GGOs on CT scan (15, 16). However, there still lack comprehensive genomic and transcriptomic studies comparing the differences between LUADs having GGO components and their counterparts not having GGO components on CT scan.

In this study, we performed whole-exome sequencing and RNA sequencing (RNA-seq) on 197 surgically resected LUADs and divided them into different groups by pathological characteristics and radiological manifestations, aiming to find key genetic factors that drive the evolution of LUAD. Twelve expression patterns were identified based on expression profiles, and pathway analysis was performed to reveal the biological functions for each pattern. Tumor intrinsic growth potential and immune microenvironment were assessed, and immune cell infiltration was calculated. Finally, to better predict tumor progression and patients’ outcome, we developed a tumor progressive (TP) index to measure the level of imbalance between tumor intrinsic growth potential and immune microenvironment and validated the index by external datasets.

## Materials and Methods

### Study Cohort

**Figure 1:**
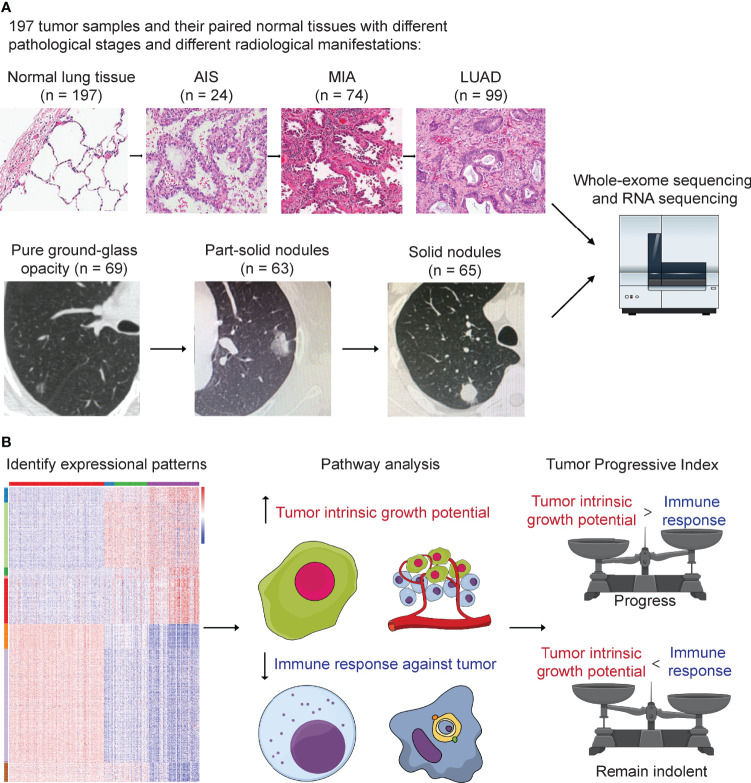
Study design. **(A)** A total number of 197 tumor samples, including 24 adenocarcinoma *in situ* (AIS), 74 minimally invasive adenocarcinoma (MIA), 99 lung adenocarcinoma (LUAD), and their paired adjacent normal lung tissue underwent whole-exome sequencing and RNA sequencing. Genomic and transcriptomic data were analyzed and compared among different groups. Samples were further divided into three groups according to their radiological manifestations: 69 pure ground-glass opacities (GGOs), 63 subsolid nodules, and 65 solid nodules. **(B)** Identification of 12 expressional patterns and development of tumor progressive index based on genomic and transcriptomic data.

A total of 197 patients with LUAD who underwent surgery between September 2011 and May 2016 at the Department of Thoracic Surgery, Fudan University Shanghai Cancer Center, were retrospectively included in this study (**Figure 1A**). None of the patients received neoadjuvant therapy. This study was approved by the Committee for Ethical Review of Research (Fudan University Shanghai Cancer Center Institutional Review Board, No. 090977-1). Informed consents of all patients for donating their samples to the tissue bank of Fudan University Shanghai Cancer Center were obtained from patients themselves or their relatives.

### Radiological and Histological Evaluation

Whole-lung CT scans were performed on each patient included before surgery as previously described (15). For each nodule, the maximum diameter of both the entire nodule and solid component on the single largest axial dimension was recorded on lung window. Pulmonary nodules were further divided into three groups: pure GGOs, where there was no solid component in one pulmonary nodule; subsolid nodules, where both solid and GGO components existed in one pulmonary nodule; and solid nodules, where the pulmonary nodule contained only solid component (**Figure 1A**). CT scans were reviewed by two radiologists independently, and interobserver and intraobserver agreements were measured to quantify the reproducibility and accuracy between the two radiologists as previously described (15).

Intraoperative frozen section diagnosis was made after the tumor was resected, and postoperative diagnosis was made after surgery by two independent pathologists. According to the IASLC/ATS/ERS classification, tumors were classified as AIS, MIA, or invasive LUAD based on their histological presentations. Invasive LUAD subtypes were further analyzed in a semi-quantitative manner, where the components of different subtypes (lepidic, acinar, papillary, micropapillary, solid, and invasive mucinous) were recorded in 5% increments. The predominant subtype was the one with the largest percentage (not necessarily 50% or higher) (17). Pathological stage of disease was determined according to the eighth TNM staging system.

### Follow-Up Protocol

Patients were followed up regularly after surgery as we previously described (15). Briefly, patients were followed up every 3 months after surgery for the first 2 years, where physical examination, chest CT scans, and abdominal ultrasonography were performed every 3 to 6 months. The follow-up interval was changed to every 6 months for the third year and once a year from then on. Brain CT or magnetic resonance imaging (MRI) and bone scintigraphy were performed every 6 months for patients with invasive adenocarcinoma in the first 3 years. In addition, positron emission tomography (PET)–CT scans were performed if necessary. Recurrence-free survival (RFS) was defined as the time between surgery and first recurrence or last follow-up. Patients with no recurrence but died from other causes were censored on that date. Overall survival (OS) was defined as the time between surgery and death or last follow-up.

### Whole-Exome Sequencing

Genomic DNA from tumors and paired adjacent normal tissues were extracted and prepared using the QIAamp DNA Mini Kit (Qiagen, Germany) following the manufacturer’s instructions. Exon libraries were constructed using the SureSelect XT Target Enrichment System. A total amount of 1–3 μg of genomic DNA for each samples was fragmented into an average size of ~200 bp. DNA was hybridized, captured, and amplified using SureSelect XT reagents and protocols to generate indexed, target-enriched library amplicons. Constructed libraries were then sequenced on the Illumina HiSeq X Ten platform and 150-bp paired-end reads were generated.

### Alignment, Mutation Calling, and Somatic Copy Number Alteration Calling

Sequence reads from the exome capture libraries were aligned to the reference human genome (hg19) using BWA-MEM (18). Picard tools were then used for marking Polymerase Chain Reaction (PCR) duplicated, and the Genome Analysis Toolkit was used to perform base quality recalibration and local indel re-alignments (19). Single-nucleotide variants (SNVs) were called using MuTect and MuTect2 (20). Indels were called using MuTect2 and Strelka v2.0.13 (21). Variantes were filtered if called by only one tool. After the variants were called, Oncotator v1.9.1 was used for annotating somatic mutations (22), and significantly mutated genes were identified using MutSig2CV (23). Tumor mutation burden (TMB) was calculated as the total number of non-synonymous SNVs and indels per sample divided by 30, given the total coverage of ~30 Mb. CNVkit v0.9.7 with default parameters was used to perform somatic copy number alteration (SCNA) analysis for alignment reads (24). Amplification and deletion peaks were identified using GISTIC2.0 from segment files (25). Amplification and deletion thresholds were set 0.1 and −0.1, respectively. Frequency distribution of amplification and deletion was shown using R package copy number v1.26.0 (26).

### RNA Sequencing and Calculation of Expression

Total RNA from tumors and paired adjacent normal tissues was extracted and prepared using NucleoZOL (Macherey-Nagel, Germany) and NucleoSpin RNA Set for NucleoZOL (Macherey-Nagel, Germany) following the manufacturer’s instructions. A total amount of 3 μg of RNA per sample was used as initial material for RNA sample preparations. Ribosomal RNA was removed using Epicenter Ribo-Zero Gold Kits (Epicenter, USA). Sequencing libraries were generated using NEBNext Ultra Directional RNA Library Prep Kit for Illumina (NEB, Ipswich, USA) according to the manufacturer’s instructions. Libraries were then sequenced on the Illumina HiSeq X Ten platform and 150-bp paired-end reads were generated. As a quality-control step, only those samples with RNA integrity number (RIN) ≥ 5.0 were included in this study.

After the data were obtained, RNA-seq reads were aligned to the reference human genome (hg19) using STAR v2.5.3 (27). Expression values were normalized to the transcripts per million (TPM) estimates using RSEM v1.3.0 (28).

### Identification of Gene Expression Patterns

One-hundred fifty samples with RIN ≥ 5.0 were divided into four groups: normal (n = 150), AIS (n = 16), MIA (n = 52), and LUAD (n = 82). Analysis of variance (ANOVA) was used to determine differentially expressed genes. First, paired comparison was performed between two adjacent groups (normal vs. AIS, AIS vs. MIA, and MIA vs. LUAD), and then, genes with p < 0.0001 and |fold change| ≥ 2 in at least one of three comparisons were considered significant and put into downstream analyses. Twelve patterns of gene expression were then identified based on their trends of expression between each two adjacent groups in the study cohort (up trend, no significant change or down trend; **Figures 1B**, **3**). Finally, KOBAS was used to perform KEGG pathway enrichment analysis for the patterns identified in the study cohort (29).

### Development of Tumor Progressive Index

TP index was developed for quantitatively measuring the level of imbalance between tumor intrinsic growth potential and immune response. To minimize the possible confounding effect introduced by genes with low expression, we first filtered out genes with mean TPM < 1.0 across all samples. Next, genes for calculating the tumor index were selected from pattern 1, where there was a significant increase between each two adjacent groups. Genes for calculating the immune index were selected from pattern 8 where there was a significant decrease from normal tissue to AIS and from MIA to LUAD. Those genes that were both selected from pattern 8 and were in an *a priori* immune-related gene list containing 730 genes were used to calculate the immune index (7). For both tumor index and immune index, first, expression for those genes for each sample was log2-transformed; then, for each sample, tumor index was calculated as the mean value of log2-transformed expression of *BCL2L15*, *COMP*, *CST1*, and *FAM83A*, whereas immune index was calculated as the mean value of log2-transformed expression of *ITLN2*, *MARCO*, *C8B*, *MASP1*, *CD36*, *TAL1*, *PPBP*, and *CDH5*. Finally, TP index was calculated as the subtraction of tumor index by immune index:

*Tumor progressive index = tumor index − immune index*

### Statistical Analysis

Clinical and pathological characteristics were recorded and compared among three groups. Pearson χ (2) test and Fisher’s exact test were used to compare categorical variables wherever applicable. Non-parametric Wilcoxon signed-rank test was used to compare medians of groups of continuous variables. Kaplan–Meier survival curves and log-rank p-values were calculated for patients’ RFS and OS. All statistical analyses and graphing work were performed using R (version 3.6.0, R Foundation for Statistical Computing, Vienna, Austria). Two-tailed p < 0.05 was considered significant for all statistical analyses unless stated otherwise.

## Results

### Pathological and Radiological Characteristics of 197 Lung Adenocarcinoma

Tumor samples were divided into three groups based on their pathological characteristics: 24 AIS, 74 MIA, and 99 LUAD, and into three groups based on their radiological manifestations: 69 pure GGOs, 63 subsolid nodules, and 65 solid nodules (**Figure 1A**). Clinical and pathological characteristics, including sex, smoking status, tumor location, pathology, and adenocarcinoma subtypes, were compared (**Table 1**). Of note, we found that there was an association between radiological and pathological presentations. Sixty-six of the 69 (95.7%) of pulmonary nodules manifesting as pure GGOs on CT scan were either AIS or MIA, whereas for pulmonary nodules manifesting as pure solid nodules, only one of 65 (1.5%) was either AIS or MIA (MIA in this case). For pulmonary nodules manifesting as subsolid nodules on CT scan, 31 of 63 (49.2%) were either AIS or MIA, and 32 (50.8%) were invasive adenocarcinoma. Compared with male patients, female patients had significantly higher frequency of pure GGO lesions (p = 0.025). For predominant adenocarcinoma subtypes, lepidic subtype was significantly enriched in the pure GGO group (p < 0.001), whereas papillary subtype was significantly enriched in the pure solid group (p = 0.004). Solid subtype was only found predominant in the pure solid group. Moreover, for presenting subtypes, lepidic subtype was significantly enriched in the pure GGO group (p = 0.001), and solid subtype was significantly enriched in the pure solid group (p = 0.014). Micropapillary subtype was also more likely to be found in the pure solid group, although without a significant difference (pure GGO vs. subsolid vs. solid: 0.0% vs. 3.1% vs. 9.4%, p = 0.472). Taken together, these results demonstrated there was a link between radiological and pathological findings; therefore, radiological manifestations can, at least partly, help predict the invasiveness of LUAD.

**Table 1:** Clinical and pathological characteristics of the study cohort (n = 197).

	Pure GGO (n = 69)	Subsolid (n = 63)	Solid (n = 65)	P-value
Sex				0.025
Female	48 (69.6%)	41 (65.1%)	31 (47.7%)	
Male	21 (30.4%)	22 (34.9%)	34 (52.3%)	
Smoking status				0.212
Former/current	16 (23.2%)	15 (23.8%)	23 (35.4%)	
Never	53 (76.8%)	48 (76.2%)	42 (64.6%)	
Tumor location				0.661
LUL	18 (26.1%)	13 (20.6%)	11 (16.9%)	
LLL	7 (10.1%)	6 (9.5%)	10 (15.4%)	
RUL	26 (37.7%)	31 (49.2%)	23 (35.4%)	
RML	7 (10.1%)	5 (7.9%)	8 (12.3%)	
RLL	11 (15.9%)	8 (12.7%)	13 (20.0%)	
Pathology				<0.001
AIS/MIA	66 (95.7%)	31 (49.2%)	1 (1.5%)	
LUAD	3 (4.3%)	32 (50.8%)	64 (98.5%)	
Predominant subtype				
Lepidic	2 (66.7%)	9 (28.1%)	3 (4.7%)	<0.001
Acinar	1 (33.3%)	17 (53.1%)	40 (62.5%)	0.453
Papillary	0 (0.0%)	6 (9.4%)	10 (15.6%)	0.004
Micropapillary	0 (0.0%)	0 (0.0%)	0 (0.0%)	–
Solid	0 (0.0%)	0 (0.0%)	9 (14.1%)	0.067
IMA	0 (0.0%)	0 (0.0%)	2 (3.1%)	0.123
Presenting subtype				
Lepidic	2 (66.7%)	12 (37.5%)	6 (9.4%)	0.001
Acinar	2 (66.7%)	24 (75.0%)	50 (78.1%)	0.863
Papillary	0 (0.0%)	9 (28.1%)	22 (34.4%)	0.407
Micropapillary	0 (0.0%)	1 (3.1%)	6 (9.4%)	0.472
Solid	0 (0.0%)	1 (3.1%)	17 (26.6%)	0.014
IMA	0 (0.0%)	0 (0.0%)	2 (3.1%)	0.572

AIS, adenocarcinoma in situ; GGO, ground-glass opacity; IAD, invasive adenocarcinoma; IMA, invasive mucinous adenocarcinoma; MIA, minimally invasive adenocarcinoma; LLL, left lower lobe; LUL, left upper lobe; RLL, right lower lobe; RML, right middle lobe; RUL, right upper lobe.

### Mutation Frequency of Tumor Suppressor Gene Increased With Tumor Progression

**Figure 2:**
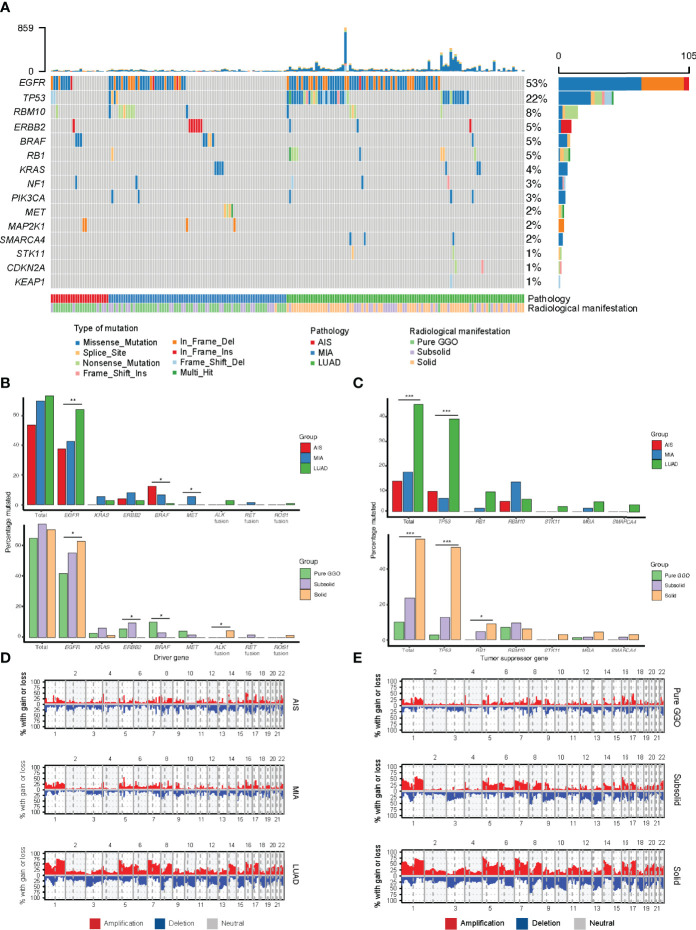
Comparison of genomic alterations for different pathological and radiological groups. **(A)** Waterfall plot showing the landscape of genomic alterations in each group. **(B)** Comparison of mutation frequency in major driver genes among different groups. **(C)** Comparison of mutation frequency in major tumor suppressor genes among different groups. **(D)** Comparison of somatic copy number alterations among different pathological groups. **(E)** Comparison of somatic copy number alterations among different radiological groups. *, p<0.05; **, p<0.01; ***, p<0.001.

We next assessed the genomic alterations of tumors with different radiological manifestations. The landscape of somatic mutations for all patients included in this study was shown in **Figure 2A**. The most frequently mutated genes in this cohort were *EGFR* (50%), followed by *TP53* (22%), *RBM10* (8%), *ERBB2* (5%), *BRAF* (5%), *RB1* (5%), *KRAS* (4%), and *NF1* (3%) (**Figure 2A**). A further comparison found that frequency of mutations in driver genes did not differ as tumor progressed, whereas frequency of mutations in tumor suppressor genes increased as tumor progressed. For individual driver genes, *EGFR* mutations were significantly more enriched in LUAD (p = 0.005), whereas *BRAF* was significantly more enriched in AIS (p = 0.028, **Supplementary Table 1**). *ALK* fusions were detected only in three LUAD samples, all of which were solid nodules on CT scan (3 of 65, p = 0.045, **Supplementary Table 2**). On the other hand, a significant difference was observed in the frequency of tumor suppressor genes. The number of mutations in common tumor suppressor genes was 2 (8.3%) for AIS, 4 (5.4%) for MIA, and 38 (38.4%) for LUAD (p < 0.001, **Supplementary Table 1**), and 7 (10.1%) for pure GGOs, 15 (23.8%) for subsolid nodules, and 37 (56.9%) for solid nodules (p < 0.001, **Supplementary Table 3**). For individual tumor suppressor genes, frequency of *TP53* mutations was found to be significantly higher in LUAD and solid nodules (p < 0.001 and p < 0.001, respectively, **Figures 2B, C** and **Supplementary Tables 1, 2**), whereas frequency of *RB1* mutations was significantly higher in solid nodules (p = 0.038) and marginally significantly higher in LUAD (p = 0.058). TMB was significantly higher in LUAD compared with AIS/MIA, and solid nodules compared with GGOs/subsolid nodules on CT scan (**Supplementary Figures 1**, **2**).

### Frequency of Somatic Copy Number Alterations Increased as Tumors Progressed

To explore the SCNAs of different stages of LUAD, we identified SCNAs from raw sequencing reads of samples of different pathological and radiological groups. Notably, the SCNA frequency including amplification and deletion increased as tumors progressed (**Figures 2D, E**). We used GISTIC2.0 to identify significant arm-level SCNAs in samples of different groups, and the union of significant arm-level event of the three groups was used for further analysis. As a result, the ratio of amplification and deletion in arm-level SCNAs increased from AIS to MIA and LUAD and from pure GGOs to subsolid and solid nodules. These results indicated that the frequency of SCNA burden was higher as tumors progressed.

### Decreased Cytotoxic CD8+ T Cells and Increased Tregs as Tumors Progressed

**Figure 3:**
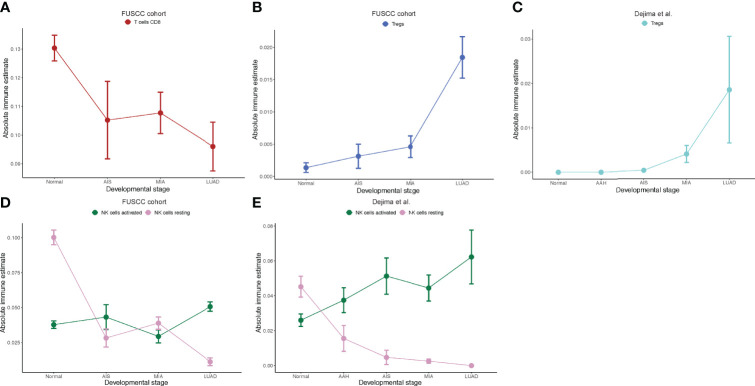
Immune cell infiltration inferred by CIBERSORT. **(A)** Prediction of number of CD8+ T cells in the Fudan University Shanghai Cancer Center (FUSCC) cohort. **(B)** Prediction of number of Tregs in the FUSCC cohort. **(C)** Prediction of number of Tregs in Dejima’s cohort. **(D)** Prediction of number of activated and resting natural killer (NK) cells in the FUSCC cohort. **(E)** Prediction of number of activated and resting NK cells in Dejima’s cohort.

To explore whether there are any differences in immune cell infiltration as tumors progress to different stages, we next used CIBERSORT, a deconvolution method to estimate the infiltration of immune cells in different pathological stages (30). We found that the number of CD8+ T cells decreased as tumor stage increased (**Figure 3A**), which was consistent with Dejima et al. on the immune evolution from neoplasia to invasive lung adenocarcionma (7). Furthermore, we found that the number of Tregs increased as tumor stage increased, suggesting an enhanced immunosuppression as disease progressed (**Figure 3B**), which was also validated by the Dejima dataset (**Figure 3C**). Interestingly, we found an increased number of activated NK cells and a decreased number of resting NK cells as disease progressed and validated this result using the Dejima dataset, suggesting that NK cells might be important in the progression of LUAD (**Figures 3D, E**). A more detailed staging of LUAD also showed similar results, with tumors at earlier stages having more CD8+ T cells and less Tregs, whereas tumors at later stages having less CD8+ T cells and more Tregs (**Supplementary Figure 3**). Taken together, our findings showed that a suppressed immune microenvironment plays an important role in the progression of LUAD.

### Expression Patterns Correlated With Tumor Intrinsic Growth Potential and Immune Function

**Figure 4:**
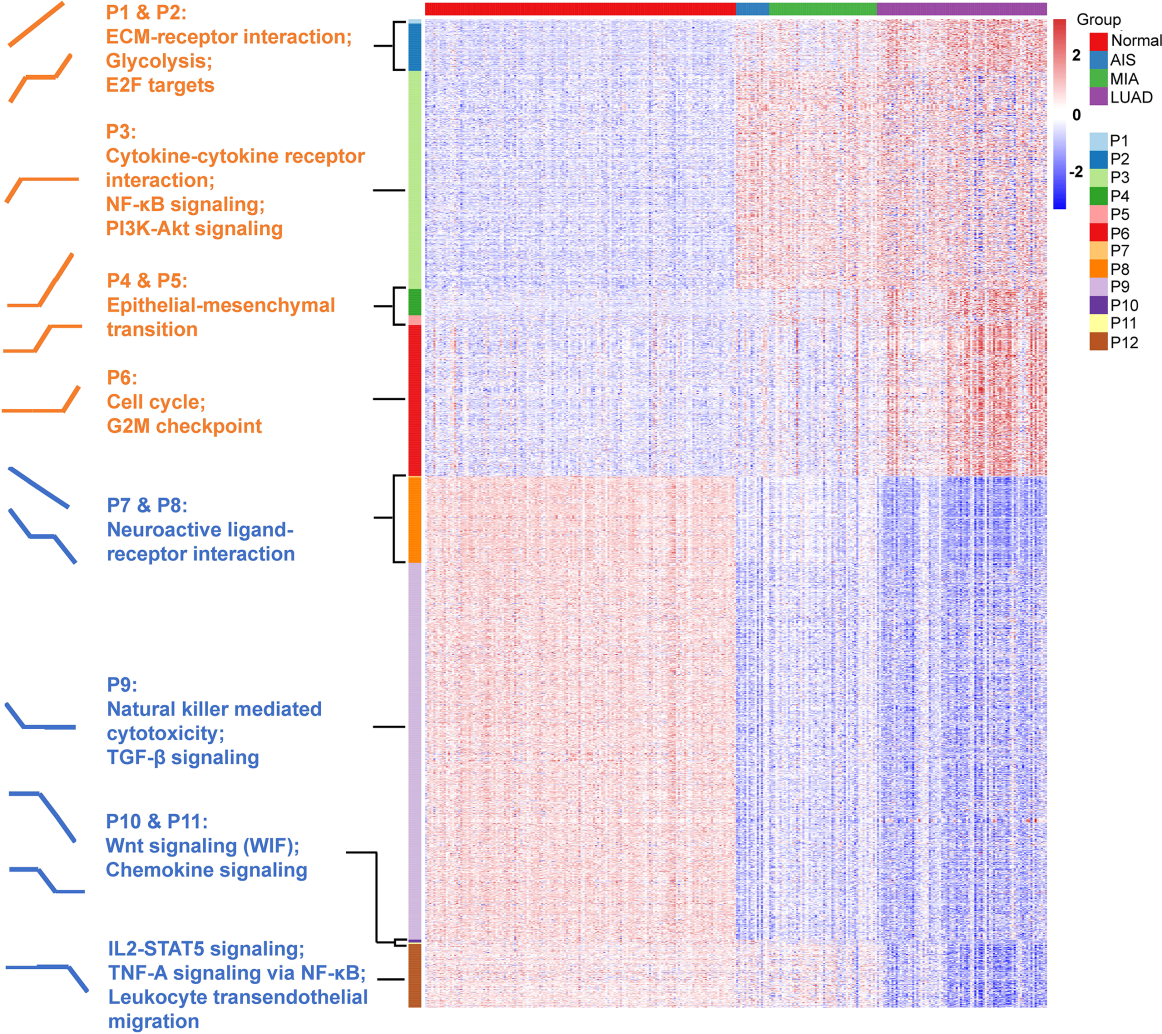
Identification of 12 expression patterns and prediction of immune cell infiltration. Pattern 1 (nine genes): increase from normal to AIS, from AIS to MIA and from MIA to LUAD; pattern 2 (97 genes): increase from normal to AIS and from MIA to LUAD, no change from AIS to MIA; pattern 3 (446 genes): increase from normal to AIS alone; pattern 4 (54 genes): no change from normal to AIS, increase from AIS to MIA and from MIA to LUAD; pattern 5 (20 genes): increase from AIS to MIA alone; pattern 6 (309 genes): increase from MIA to LUAD alone; pattern 7 (three genes): decrease from normal to AIS, from AIS to MIA and from MIA to LUAD; pattern 8 (175 genes): decrease from normal to AIS and from MIA to LUAD, no change from AIS to MIA; pattern 9 (771 genes): decrease from normal to AIS alone; pattern 10 (six genes): no change from normal to AIS, decrease from AIS to MIA and from MIA to LUAD; pattern 11 (three genes): decrease from AIS to MIA alone; pattern 12 (130 genes): decrease from MIA to LUAD alone. Gene set enrichment analysis (GSEA) was performed to assess the functional significance for those patterns.

Next, we defined expression patterns that correlated with tumor progression using RNA-seq data. One-hundred fifty samples with RIN ≥ 5.0 were included in this part. We developed an ANOVA model with pathological stage (normal tissue, AIS, MIA, and LUAD) as a factor and patient as a random effect. A total of 2023 genes with p < 0.0001 and |fold change| > 2 in pairwise comparisons of two adjacent groups of patients (normal tissue vs. AIS, AIS vs. MIA, and MIA vs. LUAD, respectively) were divided into 12 following patterns: pattern 1 (nine genes): increase from normal to AIS, from AIS to MIA and from MIA to LUAD; pattern 2 (97 genes): increase from normal to AIS and from MIA to LUAD, no change from AIS to MIA; pattern 3 (446 genes): increase from normal to AIS alone; pattern 4 (54 genes): no change from normal to AIS, increase from AIS to MIA and from MIA to LUAD; pattern 5 (20 genes): increase from AIS to MIA alone; pattern 6 (309 genes): increase from MIA to LUAD alone; pattern 7 (three genes): decrease from normal to AIS, from AIS to MIA and from MIA to LUAD; pattern 8 (175 genes): decrease from normal to AIS and from MIA to LUAD, no change from AIS to MIA; pattern 9 (771 genes): decrease from normal to AIS alone; pattern 10 (6 genes): no change from normal to AIS, decrease from AIS to MIA and from MIA to LUAD; pattern 11 (three genes): decrease from AIS to MIA alone; pattern 12 (130 genes): decrease from MIA to LUAD alone (**Figure 4** and **Supplementary Table 3**). Pathway enrichment analysis showed that pathways associated with tumor invasiveness and cell growth were enriched in up-trend patterns (patterns 1 to 6). Of note, Phosphatidylinositol 3-Kinase - Protein Kinase B (PI3K-AKT) signaling pathway and NF-κB signaling pathway were upregulated in AIS compared with normal tissue, suggesting that malignant behaviors could exist in as early as AIS. Epithelial–mesenchymal transition, which was highly associated with tumor metastasis, was also found to be increased from AIS to MIA, suggesting an acquisition of metastatic potential as invasive subtypes of LUAD emerge (31). Moreover, cell cycle was found to be increased from MIA to LUAD, consistent with the fact that AIS/MIA behaved more indolent than LUAD. On the other hand, for down-trend patterns, natural killer–mediated cytotoxicity and Transforming Growth Factor-beta (TGF- β) signaling pathway were found to be decreased from normal lung tissue to AIS, suggesting an inhibited immune response against tumor. Chemokine signaling, Interleukin2-Signal Transducer and Activator of Transcription 5 (IL2-STAT5) signaling, TNF-A signaling *via* NF-κB and leukocyte transendothelial migration were also found to be enriched in down-trend patterns, further suggesting an impaired immune function in tumor microenvironment. Interestingly, Wnt signaling pathway, which was often upregulated in cancer, was found to be decreased from AIS to MIA and from MIA to LUAD. A deeper look into pattern 10 showed that this finding was contributed by WIF1 (Wnt inhibiting factor 1), which was an inhibitor of Wnt signaling pathway. Taken together, our results suggest that both an increased tumor intrinsic growth potential and an inhibited immune microenvironment contributed to the development and progression of LUAD.

### TP Index Measured the Imbalance Between Tumor Intrinsic Growth Potential and Immune Microenvironment

**Figure 5:**
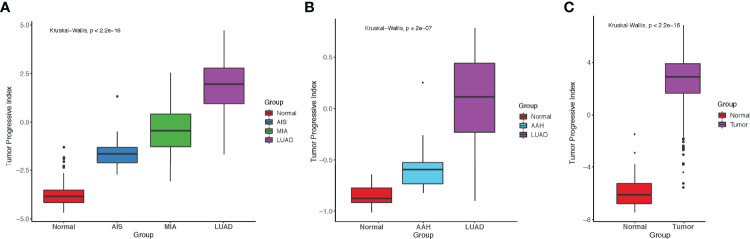
Comparison of tumor progressive index for different datasets. **(A)** Boxplot showing the index increased as tumor progressed in the FUSCC cohort. **(B)** Boxplot showing the index increased as tumor progressed in Dejima’s cohort. **(C)** Boxplot showing the difference of tumor progressive index between normal and tumor samples in the TCGA-LUAD cohort.

To better predict tumor progression and outcome of patients, TP index were built to measure the imbalance between tumor intrinsic growth potential and immune microenvironment. On the basis of our previous findings, we used the genes in pattern 1 and immune-related genes in patterns 7 and 8 with mean TPM ≥ 1 across all samples (methods). Four genes that were associated with apoptosis, tumor metastasis and progression (*BCL2L15*, *COMP*, *CST1*, and *FAM83A*) reflected tumor intrinsic growth potential, whereas eight genes (*ITLN2*, *MARCO*, *C8B*, *MASP1*, *CD36*, *TAL1*, *PPBP*, and *CDH5*) that were associated with immune microenvironment reflected immune response against tumors. A negative TP index indicates that the immune system is competent enough to suppress the progression of tumors, whereas a positive TP index indicates that the immune system can no longer suppress the growth of tumor cells. In our study cohort, TP index was negative in normal tissues but positive in AIS and stages onward, indicating that immune escape already existed in AIS, the precursor stage of LUAD, and became more severe with the progression of disease (**Figure 5A**). Same increasing trend was observed in another dataset, which showed a significant increase of TP index from normal tissue to atypical adenomatous hyperplasia (AAH) then to LUAD (**Figure 5B**) (32). Interestingly, there was a negative TP index in the stage of AAH, where tumors could not overcome the immune system to metastasize further. Although the TCGA-LUAD dataset does not contain pre-invasive stages of LUAD, we still calculated the TP index for each sample and compared it between normal and tumor samples. Not surprisingly, tumor samples had significantly higher TP index than normal samples (**Figure 5C**).

### TP Index Effectively Predicted the Prognosis in Patients With Lung Adenocarcinoma

**Figure 6:**
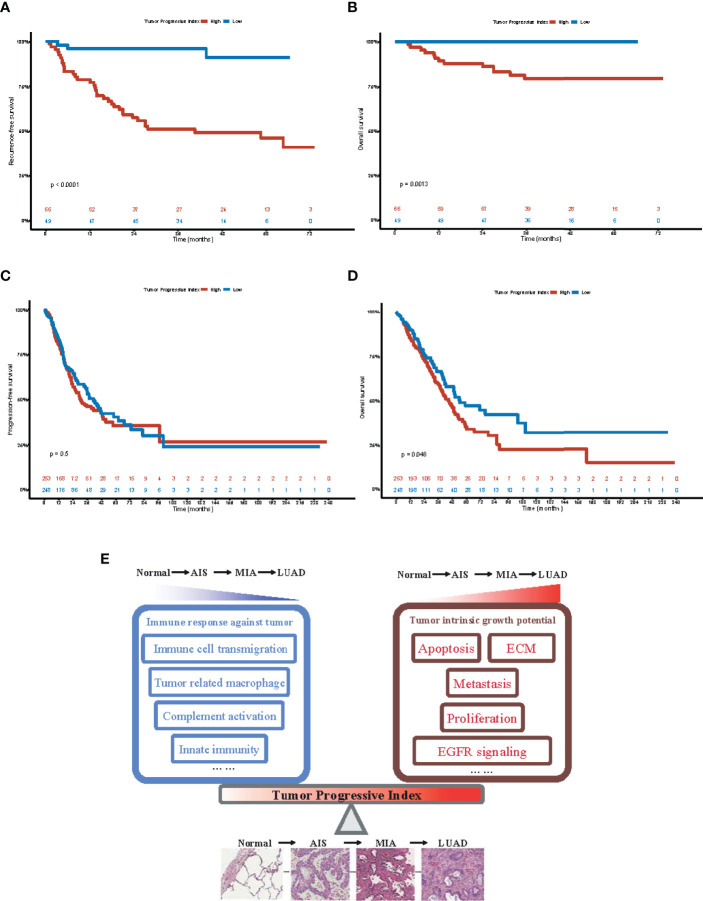
Prognostic value of tumor progressive index for patients with lung adenocarcinoma. **(A)** Recurrence-free survival for patients with tumor progressive index higher and lower than the median value across the Fudan University Shanghai Cancer Center (FUSCC) cohort. **(B)** Overall survival for patients with tumor progressive index higher and lower than the median value across the Fudan University Shanghai Cancer Center (FUSCC) cohort. **(C)** Recurrence-free survival for patients with tumor progressive index higher and lower than the median value across the TCGA-LUAD cohort. **(D)** Overall survival for patients with tumor progressive index higher and lower than the median value across the TCGA-LUAD cohort. **(E)** Schematic demonstration showing the imbalance between tumor intrinsic growth potential and immune response against tumor, which can be measured by tumor progressive index, leads to the evolution and progression of lung adenocarcinoma.

To investigate whether our TP index had prognostic value for patients with LUAD, survival analysis was performed on both our dataset and TCGA-LUAD dataset. Our results showed that in our dataset, patients with a high TP index had poorer RFS (HR, 10.47; 95% CI, 3.21–34.14) and OS (HR, 4.83e8; 95% CI, 0–Inf; **Figures 6A, B**), whereas in the TCGA-LUAD dataset, patients with a higher TP index had poorer OS (HR, 1.35; 95% CI, 1.00–1.81) but progression-free survival (PFS) was comparable for the two groups (HR, 1.10; 95% CI, 0.83–1.45; **Figures 6C, D**). Taken together, our results suggested that increased tumor intrinsic growth potential and impaired immune response against tumor work together to drive the progression of LUAD, and our TP index, which measures the level of imbalance between tumor intrinsic growth potential and tumor immune microenvironment, is of prognostic value for patients with LUAD (**Figure 6E**).

## Discussion

In this study, we provided a comprehensive analysis integrating clinical, radiological, pathological, genomic, and transcriptomic analysis of 197 pulmonary lesions with different radiological and pathological manifestations.

We first assessed the transcriptomic profiles of our cohort. On the basis of the differentially expressed genes between each two adjacent groups, 12 expression patterns were identified (**Figure 4**). Genes associated with cell cycle and G2M checkpoint were found to be significantly upregulated from MIA to LUAD, but not different from normal tissue to MIA. This indicates that pre-invasive stages of LUAD had more indolent behaviors, which might explain why AIS and MIA had a nearly 100% 5-year survival rate after surgical resection. Histologically, AIS is defined as a ≤3-cm adenocarcinoma lacking invasive patterns, whereas MIA is a ≤3-cm adenocarcinoma with invasive patterns of no more than 5 mm in size (31). PI3K-AKT signaling pathway, a classical pathway that was upregulated in various cancer types, was found to be increased from normal tissue to AIS (33). Although AIS is the precursor of LUAD, this finding shows that cells in this stage already have an increased potential for growth. On the other hand, we found that epithelial–mesenchymal transition, a biological process known to increase metastatic potential of cancer cells (34), was found to be significantly increased from AIS to MIA, consistent with histological differences between AIS and MIA. Although AIS and MIA both have a nearly perfect prognosis, our results suggest that MIA should be surgically intervened before it progresses to the next stage as invasive patterns already exist. To our surprise, several immune-related pathways were found in down-trend expression patterns, indicating a decreased or impaired immune response as tumors progress. Consistent with this finding, using CIBERSORT to deconvolute our bulk-sequencing data, we found a decrease in the number of CD8+ cytotoxic T cells and an increase in the number of Treg cells, which was also validated by another dataset that compared immune cells among normal tissue, AAH, AIS, MIA, and LUAD (7). Taken together, our results demonstrated that increased tumor intrinsic growth signals and decreased immune response orchestrate the evolution of LUAD.

We next demonstrated that there was an association between radiological and pathological presentations. Most nodules that were GGOs on CT scan were AIS or MIA, whereas most solid nodules were invasive LUAD (**Table 1**). This might provide explanation of why pulmonary nodules manifesting as GGOs on CT scan usually have indolent clinical courses. This result was also consistent with previous studies and provided a link between histological and radiological manifestations (35, 36). For major driver mutations, we found that frequency of neither *EGFR* nor *KRAS* mutations was significantly different among the three groups (**Figure 2B** and **Supplementary Table 2**). *BRAF* mutations were more enriched in the pure GGO group, whereas *ALK* fusion was only seen in the solid group. On the other hand, the frequency of mutations in tumor suppressor genes was significantly different among the three groups, with a sharp increase from 2 (2.9%) in the pure GGO group, to 8 (12.7%) in the subsolid group, and 33 (50.8%) in the solid group (**Figure 2C** and **Supplementary Table 2**), suggesting a pivotal role that tumor suppressor genes play in the progression of LUAD. For individual tumor suppressor genes, *TP53* and *RB1* were significantly different among the three groups (p < 0.001 and p = 0.038, respectively; **Figure 2C** and **Supplementary Table 2**).

Previous studies have discussed the genomic alterations in pulmonary nodules manifesting as GGO. In 2015, Kobayashi et al. evaluated *EGFR*, *KRAS*, *ALK*, and *HER2* mutations in 104 pulmonary nodules, manifesting as GGO. They found that *EGFR* was mutated in 64% of all the 104 samples, whereas *KRAS*, *ALK*, and *HER2* were mutated in 4%, 3%, and 4% of the samples, respectively (37). In 2018, Lu et al. reported that *EGF*R was mutated in 75 of 156 (48.1%) patients with LUAD, which was similar to 50% in our cohort (38). We also observed a significant difference in *EGFR* mutation frequency among three radiological groups (**Figure 2B** and **Supplementary Table 2**). In 2020, Li et al. performed whole-exome sequencing on 154 pulmonary subsolid nodules from 120 patients and found that *EGFR* was the most frequently mutated gene, followed by *RBM10*, *TP53*, *STK11*, and *KRAS*. They also found that frequency of *EGFR*, *RBM10*, and *TP53* was significantly different between pure GGO and subsolid nodules (39). Although not significantly different for *EGFR* and *RBM10* in our cohort, there was indeed a significant difference of the frequency of *TP53* mutations (**Figure 2C** and **Supplementary Table 2**).

For SCNAs, we found that the frequency of SCNAs increased as tumors progressed. As the solid components increased, the genome became more unstable. Frequency of arm-level alterations also followed the same trend, where tumors at earlier stages and manifested as pure GGOs on CT scan had fewer significant arm-level events, whereas tumors at later stages and manifested as solid nodules had more significant arm-level events. Genomic instability was widely reported to be associated with increased rate of tumor proliferation and progression among different cancer types (40–42).

Tumor intrinsic growth potential and immune microenvironment are both associated with tumor evolution and progression (43, 44). On the basis of our findings, a TP index was designed to quantitatively measure the level of imbalance between tumor intrinsic growth potential and immune microenvironment. Interestingly, AAH, a stage of pre-cancerous hyperplasia, had a negative TP index, indicating some key alteration would be needed to activate the malignant transformation of cells. AIS, MIA, and LUAD all had a progressive index of more than 0, and the index increased as the tumor developed to the next stage. Furthermore, patients with a high TP index tended to have a poorer RFS and OS. Taken together, our data demonstrated that TP index can be used as a predictor for disease progression and prognosis, discovering new markers for predicting patients’ survival and potential drug targets.

In our study, surgically resected specimens were used. However, in some cases, cytology is the only available specimens. Previous studies have shown that performing next-generation sequencing on cytological samples can yield comparable results than on histological samples (45, 46). In the context of lung transplantation, donors with suspected tumors are rarely used due to the risk of transmission (47). However, as patients needing lung transplantation are increasing and pulmonary nodules are increasingly diagnosed, pulmonary nodules that are not malignant need to be excluded from the blacklist. A fast assessment based on pathology and genomics using cytological specimens might be a possible solution, shedding light on future application of molecular testing.

In summary, this study integrates the genomic alterations, transcriptomic profiles, and histological and radiological progression of LUAD, providing deeper understandings of the evolution of this disease.

## Data Availability Statement

The datasets presented in this study can be found in online repositories. The names of the repository/repositories and accession number(s) can be found below: European Genome-phenome Archive (EGA). Accession number: EGAS00001004006 ("https://ega-archive.org/studies/EGAS00001004006)". Source data and codes for generating the figures in this study are available at https://github.com/yuezhao97/Progression_LUAD_project.

## Ethics Statement

The studies involving human participants were reviewed and approved by the Committee for Ethical Review of Research (Fudan University Shanghai Cancer Center Institutional Review Board No. 090977-1). The patients/participants provided their written informed consent to participate in this study.

## Author Contributions

HC, LS, ZC, and YuZ designed the study. YuZ, JS, and JG performed data analysis. YuZ, HH, and ZG performed experiments. QZ and YL reviewed pathological slides. YY, TY, FF, CD, ZM, YaZ, DZ, and SZ collected samples. JS and LS helped with quality control of sequencing data. YuZ, JS, and JG drafted the manuscript. All authors contributed to the article and approved the submitted version.

## Funding

The design of the study and collection, analysis, and interpretation of data and in writing the manuscript were supported by the National Natural Science Foundation of China (81930073), Shanghai Science and Technology Innovation Action Project (20JC1417200), Shanghai Municipal Science and Technology Major Project (2017SHZDZX01 and VBH1323001/026), Shanghai Municipal Key Clinical Specialty Project (SHSLCZDZK02104), Pilot Project of Fudan University (IDF159045), Shanghai Sailing Program (22YF1408900), and Project supported by Shanghai Municipal Science and Technology Major Project (2017SHZDZX01).

## Conflict of Interest

The authors declare that the research was conducted in the absence of any commercial or financial relationships that could be construed as a potential conflict of interest.

## Publisher’s Note

All claims expressed in this article are solely those of the authors and do not necessarily represent those of their affiliated organizations, or those of the publisher, the editors and the reviewers. Any product that may be evaluated in this article, or claim that may be made by its manufacturer, is not guaranteed or endorsed by the publisher.

## Supplementary Material

Supplementary Figure 1 Comparison of tumor mutation burden (TMB) for different pathological groups.

Supplementary Figure 2 Comparison of tumor mutation burden (TMB) for different radiological groups.

Supplementary Figure 3 Prediction of number of **(A)** CD8+ T cells, **(B)** Tregs, and **(C)** natural killer (NK) cells for different tumor stages.
