# Supplementary figures and images for: Increased Tumor Intrinsic Growth Potential and Decreased Immune Function Orchestrate the Progression of Lung Adenocarcinoma
Source: Front Immunol. 2022 Jul 1;13:921761. doi: 10.3389/fimmu.2022.921761 (PMC9283781; doi:10.3389/fimmu.2022.921761)

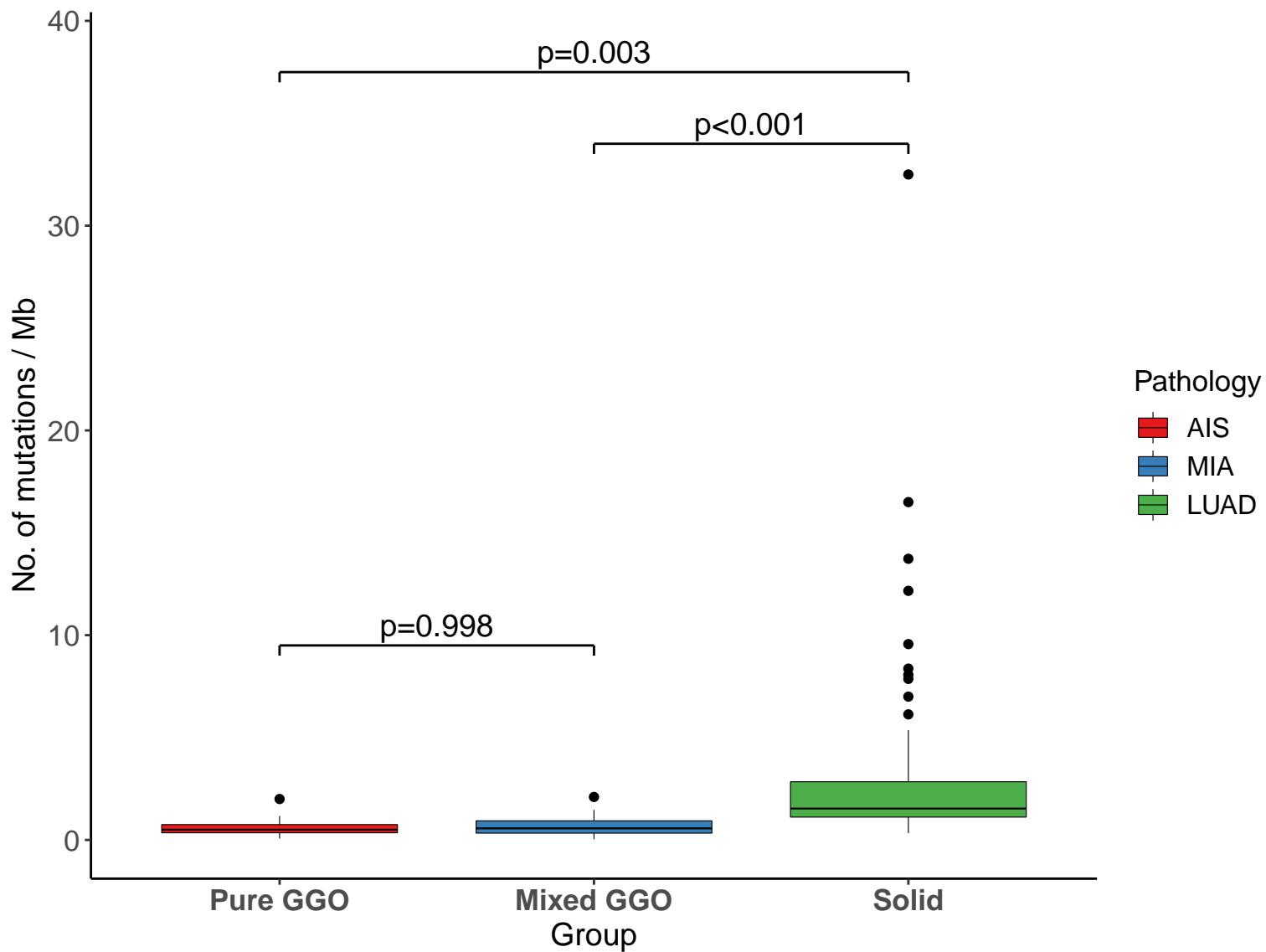

Supplement: Supplementary Figure 1 — Comparison of tumor mutation burden (TMB) for different pathological groups. [file Image_1.pdf]

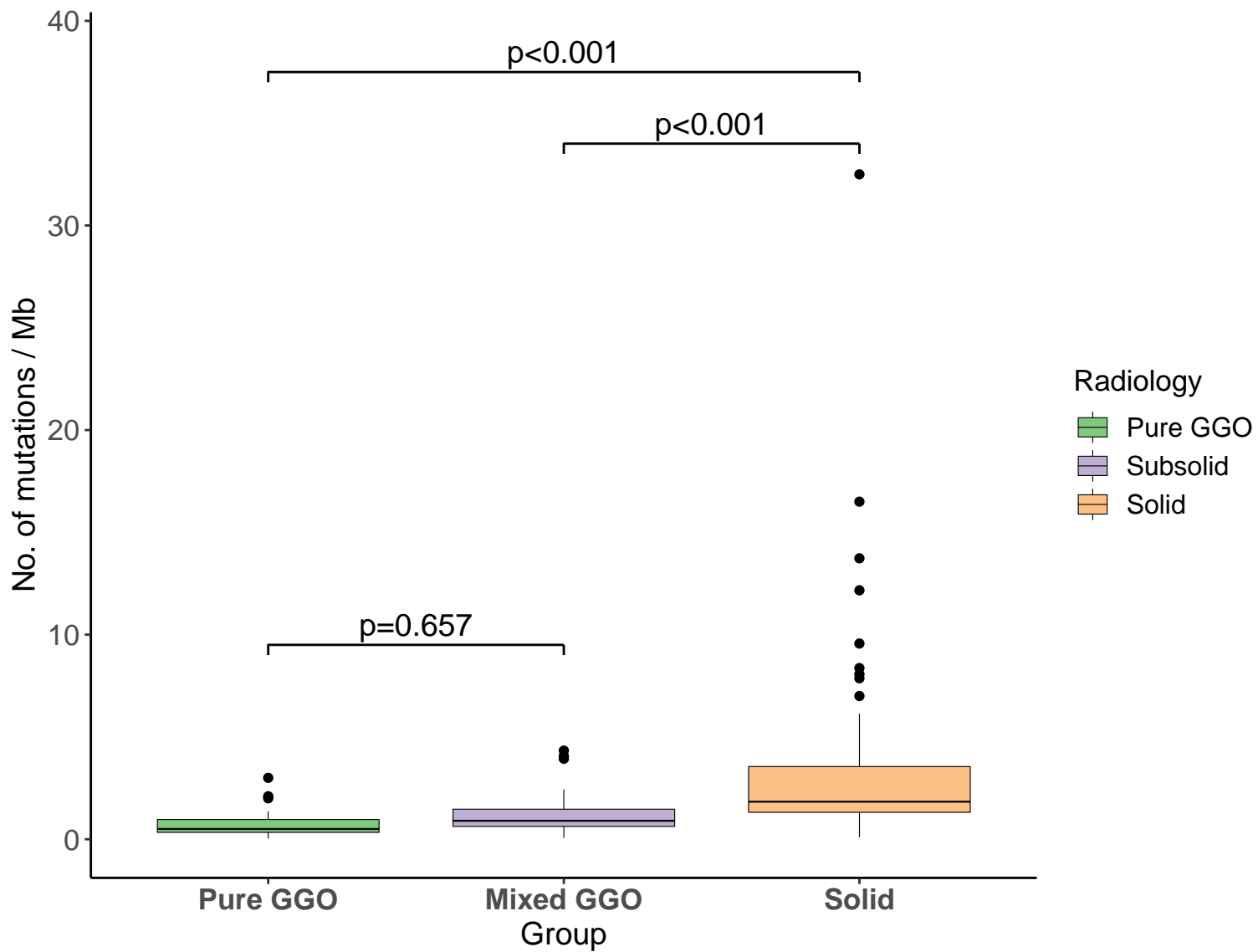

Supplement: Supplementary Figure 2 — Comparison of tumor mutation burden (TMB) for different radiological groups. [file Image_2.pdf]

A

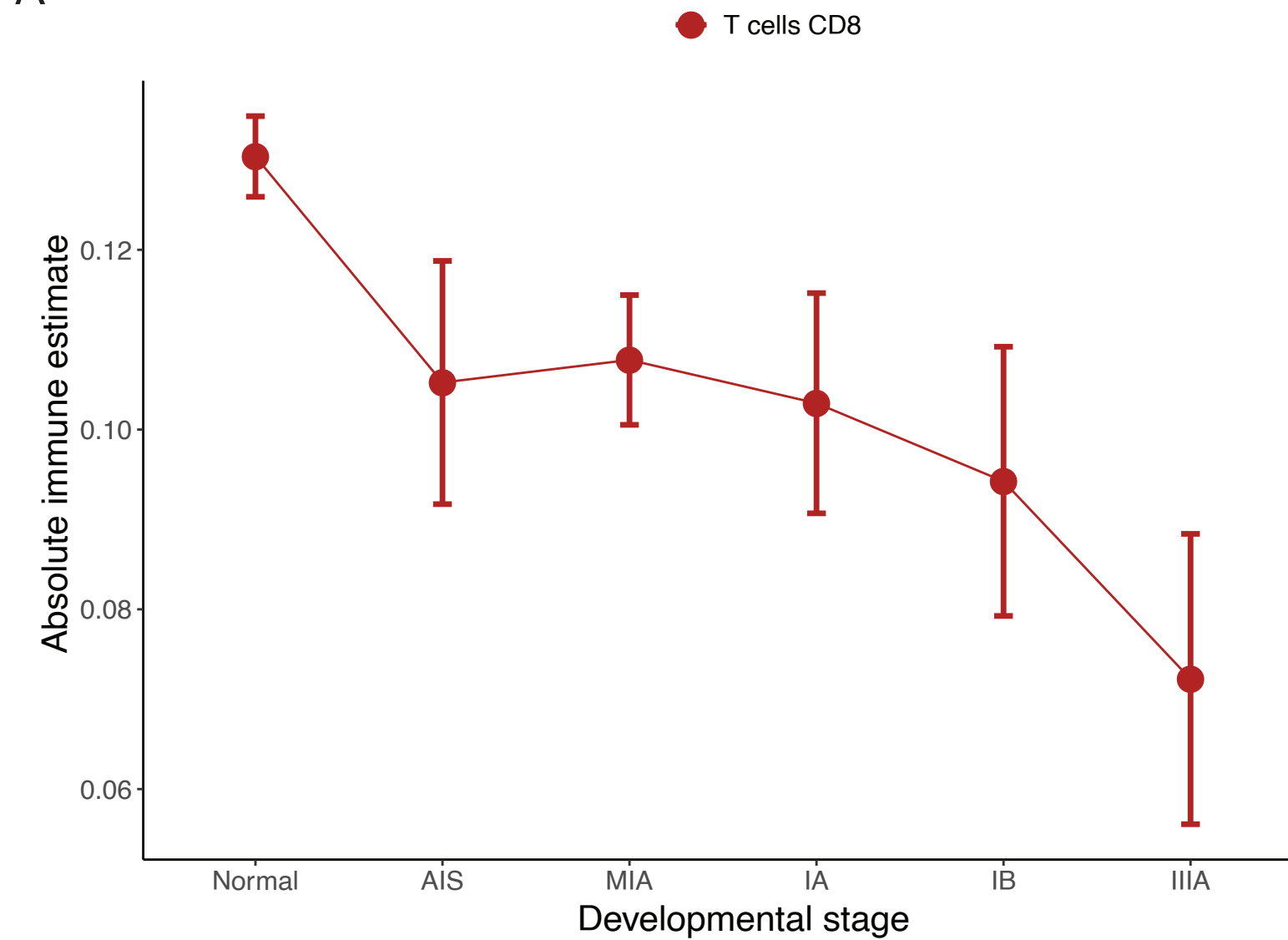

B

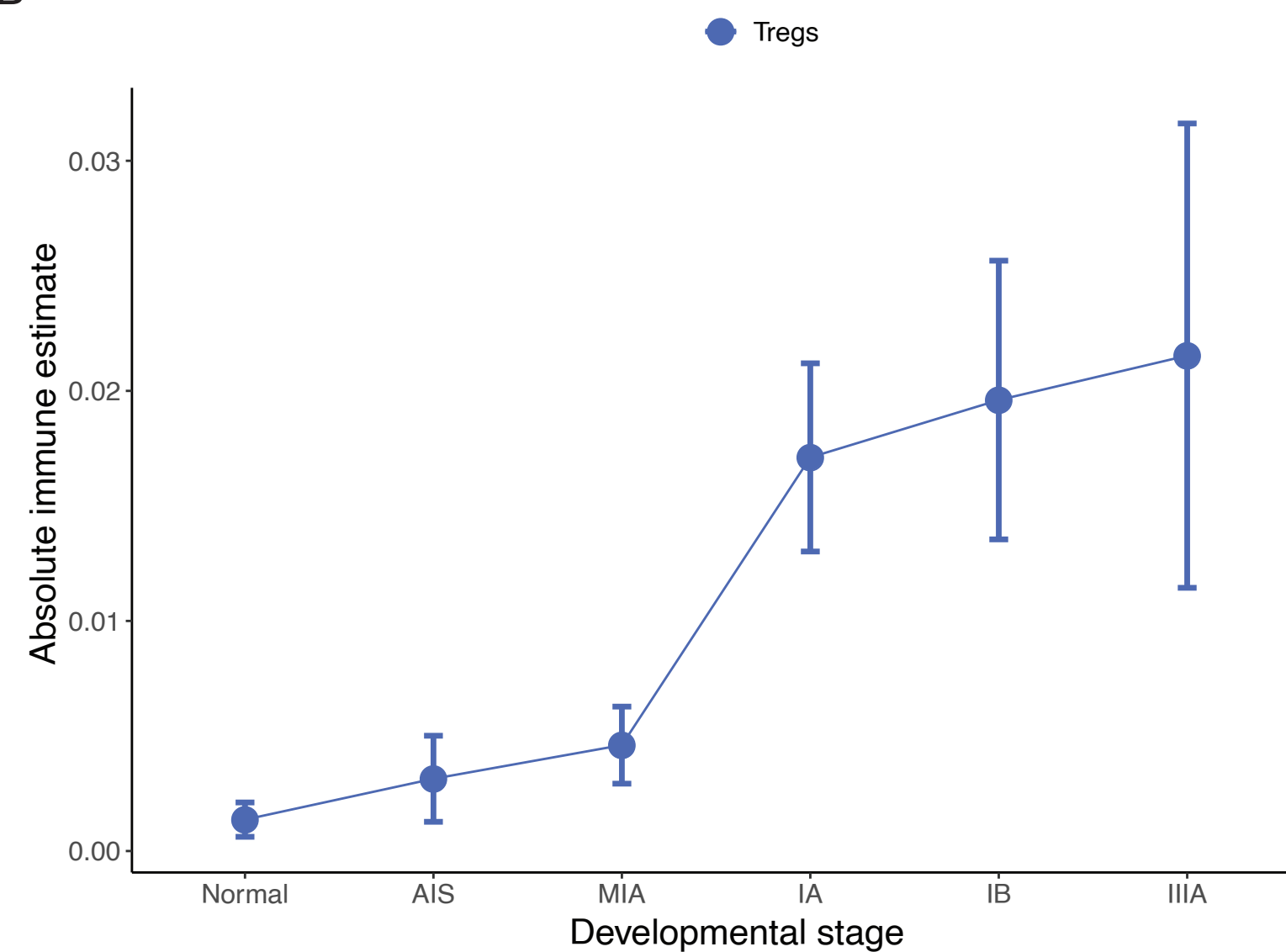

C

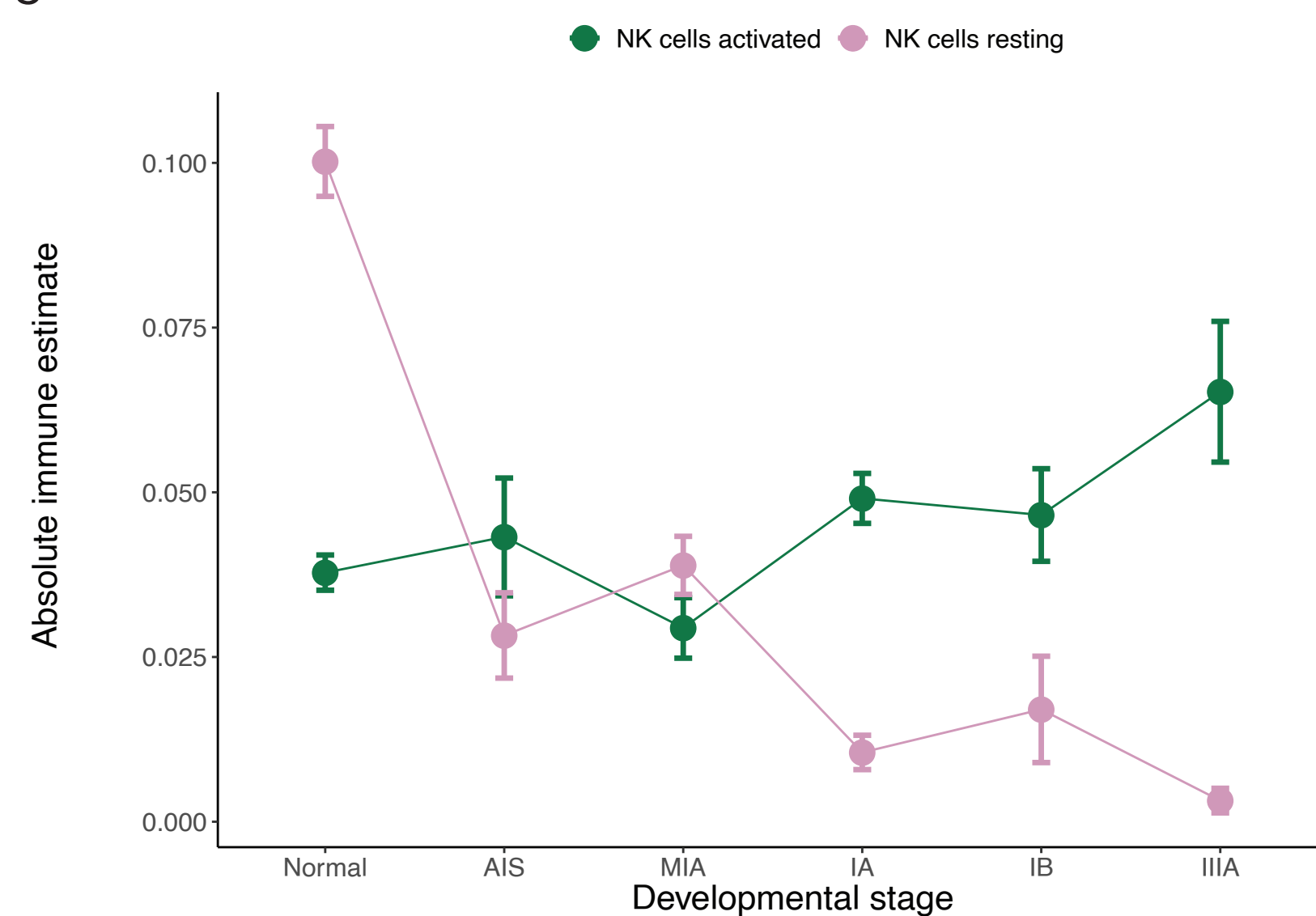

Supplement: Supplementary Figure 3 — Prediction of number of (A) CD8+ T cells, (B) Tregs, and (C) natural killer (NK) cells for different tumor stages. [file Image_3.pdf]
