# Supplementary material for: Increased Tumor Intrinsic Growth Potential and Decreased Immune Function Orchestrate the Progression of Lung Adenocarcinoma
Source: Front Immunol. 2022 Jul 1;13:921761. doi: 10.3389/fimmu.2022.921761 (PMC9283781; doi:10.3389/fimmu.2022.921761)
Supplement: Supplementary file 4 [file Table_1.docx]

|  | AIS  (n = 24) | MIA  (n = 74) | LUAD  (n = 99) | P-value |
| --- | --- | --- | --- | --- |
| Driver genes | 13 (54.2%) | 51 (68.9%) | 73 (73.7%) | 0.171 |
| *EGFR* | 9 (37.5%) | 32 (43.2%) | 64 (64.6%) | 0.005 |
| *KRAS* | 0 (0.0%) | 4 (5.4%) | 3 (3.0%) | 0.427 |
| *ERBB2* | 1 (4.2%) | 6 (8.1%) | 3 (3.0%) | 0.315 |
| *BRAF* | 3 (12.5%) | 5 (6.8%) | 1 (1.0%) | 0.028 |
| *MET* | 0 (0.0%) | 4 (5.4%) | 0 (0.0%) | 0.034 |
| *ALK* fusion | 0 (0.0%) | 0 (0.0%) | 3 (3.0%) | 0.221 |
| *RET* fusion | 0 (0.0%) | 1 (1.4%) | 0 (0.0%) | 0.434 |
| *ROS1* fusion | 0 (0.0%) | 0 (0.0%) | 1 (1.0%) | 0.608 |
| Tumor suppressor genes | 3 (12.5%) | 12 (16.2%) | 44 (44.4%) | < 0.001 |
| *TP53* | 2 (8.3%) | 4 (5.4%) | 38 (38.4%) | < 0.001 |
| *RB1* | 0 (0.0%) | 1 (1.4%) | 8 (8.1%) | 0.058 |
| *RBM10* | 1 (4.2%) | 9 (12.2%) | 5 (5.1%) | 0.173 |
| *STK11* | 0 (0.0%) | 0 (0.0%) | 2 (2.0%) | 0.368 |
| *MGA* | 0 (0.0%) | 1 (1.4%) | 4 (4.0%) | 0.377 |
| *SMARCA4* | 0 (0.0%) | 0 (0.0%) | 3 (3.0%) | 0.221 |

Supplementary table 1. Mutational status of major driver and tumor suppressor genes in the study cohort (n = 197).

Abbreviations: AIS, adenocarcinoma *in situ*; MIA, minimally invasive adenocarcinoma; LUAD, lung adenocarcinoma.
