# Supplementary material for: Increased Tumor Intrinsic Growth Potential and Decreased Immune Function Orchestrate the Progression of Lung Adenocarcinoma
Source: Front Immunol. 2022 Jul 1;13:921761. doi: 10.3389/fimmu.2022.921761 (PMC9283781; doi:10.3389/fimmu.2022.921761)
Supplement: Supplementary file 5 [file Table_2.docx]

|  | Pure GGO  (n = 69) | Subsolid  (n = 63) | Solid  (n = 65) | P-value |
| --- | --- | --- | --- | --- |
| Driver genes | 45 (65.2%) | 47 (74.6%) | 46 (70.8%) | 0.495 |
| *EGFR* | 29 (42.0%) | 35 (55.6%) | 41 (63.1%) | 0.046 |
| *KRAS* | 2 (2.9%) | 4 (6.3%) | 1 (1.5%) | 0.318 |
| *ERBB2* | 4 (5.8%) | 6 (9.5%) | 0 (0.0%) | 0.046 |
| *BRAF* | 7 (10.1%) | 2 (3.2%) | 0 (0.0%) | 0.016 |
| *MET* | 3 (4.3%) | 1 (1.6%) | 0 (0.0%) | 0.195 |
| *ALK* fusion | 0 (0.0%) | 0 (0.0%) | 3 (4.6%) | 0.045 |
| *RET* fusion | 0 (0.0%) | 1 (1.6%) | 0 (0.0%) | 0.343 |
| *ROS1* fusion | 0 (0.0%) | 0 (0.0%) | 1 (1.5%) | 0.360 |
| Tumor suppressor genes | 7 (10.1%) | 15 (23.8%) | 37 (56.9%) | < 0.001 |
| *TP53* | 2 (2.9%) | 8 (12.7%) | 34 (52.3%) | < 0.001 |
| *RB1* | 0 (0.0%) | 3 (4.8%) | 6 (9.2%) | 0.038 |
| *RBM10* | 5 (7.2%) | 6 (9.5%) | 4 (6.2%) | 0.765 |
| *STK11* | 0 (0.0%) | 0 (0.0%) | 2 (3.1%) | 0.129 |
| *MGA* | 1 (1.4%) | 1 (1.6%) | 3 (4.6%) | 0.429 |
| *SMARCA4* | 0 (0.0%) | 1 (1.6%) | 2 (3.1%) | 0.347 |

Supplementary table 2. Mutational status of major driver and tumor suppressor genes in the study cohort (n = 197).
